# Serum fibroblast growth factor 23 and kidney injury molecule-1 in the prediction of acute kidney injury in critically-ill patients

**DOI:** 10.1080/0886022X.2025.2575918

**Published:** 2025-10-28

**Authors:** Lina Zhang, Limeng Wang, Baolan Yan, Xiaohang Pei, Ning Wang, Lei Yan, Huixia Cao, Fengmin Shao

**Affiliations:** a Department of Nephrology, Henan Provincial Key Laboratory of Kidney Disease and Immunology, Henan Provincial Clinical Research Center for Kidney Disease, Henan Provincial People’s Hospital and People’s Hospital of Zhengzhou University, Zhengzhou, Henan, China; b Department of Hematology, Henan Provincial People’s Hospital and People’s Hospital of Zhengzhou University, Zhengzhou, Henan, China

**Keywords:** Acute kidney injury, fibroblast growth factor 23, kidney injury molecule-1, diagnosis, severity, renal function recovery

## Abstract

This study aimed to evaluate the value of serum intact fibroblast growth factor 23 (iFGF23), C-terminal FGF23 (cFGF23), and kidney injury molecule-1 (KIM-1) in predicting acute kidney injury (AKI) onset, severity, and renal function recovery in critically ill patients. A prospective cohort of 96 adults admitted to the intensive care unit (ICU) was analyzed. Patients were stratified into AKI (*n* = 51) and non-AKI (*n* = 45) groups based on KDIGO criteria. Serum iFGF23, cFGF23, and KIM-1 levels were measured at ICU admission. All three biomarkers were significantly elevated in AKI group compared to non-AKI group (all *p* < 0.01). Receiver operating characteristic (ROC) analysis showed that KIM-1 had the highest accuracy with an area under the curve (AUC) of 0.924 [95% confidence interval (CI) 0.866–0.983], followed by cFGF23 with an AUC of 0.779 (95% CI 0.676–0.881) and iFGF23 with an AUC of 0.672 (95% CI 0.554–0.789). Both iFGF23 (AUC 0.793, 95% CI 0.650–0.935) and cFGF23 (AUC 0.746, 95% CI 0.593–0.899) effectively predicted severe AKI (stages 2–3, *n* = 25). In contrast, KIM-1 showed no discriminative capacity (*p* > 0.05). ROC analysis indicated that none of the three biomarkers could predict renal function recovery (*p* > 0.05). Notably, KIM-1 may serve as a highly accurate marker for early AKI diagnosis, whereas serum FGF23 appears more promising for evaluating AKI severity. These complementary roles highlight the potential value of combining biomarkers to improve risk stratification in critically ill patients.

## Introduction

Acute kidney injury (AKI) is a prevalent and serious issue among patients in hospitals, marked by a rapid decline in renal function. It not only affects patient survival but may also impact long-term prognosis and quality of life, thus placing a significant burden on families and the healthcare system. According to epidemiological data, AKI affects approximately 12% of general hospitalizations [1] and more than half of intensive care unit (ICU) patients [2]. Researches indicated that AKI mortality in hospitalized patients range from 8.8% to 35.3% [3], whereas in ICU patients, the rate can reach 69.6% [3]. Thus, identifying AKI early and intervening promptly are crucial for improving its prognosis.

AKI is currently diagnosed based on increased serum creatinine (Scr) levels and/or reduced urine output. However, Scr level is easily affected by various confounding factors, including age, diet, and medications, while urine output remains an insensitive and imprecise measure [4,5]. These limitations may lead to delayed AKI diagnosis and subsequent therapeutic intervention. Accordingly, there is a critical need to develop biomarkers with greater sensitivity and specificity for the early prediction of AKI.

Several novel biomarkers, including fibroblast growth factor-23 (FGF-23) and kidney injury molecule-1 (KIM-1), have shown promise in predicting early AKI before a significant rise in Scr. FGF23, a phosphatonin primarily secreted by osteocytes and osteoblasts [6,7], rises rapidly in AKI and has been associated with adverse outcomes [8,9]. FGF23 can be measured by two assays: intact FGF23 (iFGF23), which measures the biologically active full-length hormone, and C-terminal FGF23 (cFGF23), which recognizes both the intact hormone and C-terminal fragments [10]. Because these assays interrogate different facets of FGF23 biology, they may exhibit distinct associations across clinical contexts. KIM-1, a 38.7-kDa type I transmembrane glycoprotein, is barely expressed in normal kidneys but is highly upregulated in AKI patients [11]. Several studies have suggested KIM-1 as a sensitive and specific biomarker of AKI and a potential predictor of outcomes [12].

Despite growing interest, comparisons of serum iFGF23 vs. cFGF23 in ICU populations remain limited, particularly when evaluated in parallel for early AKI diagnosis, severity stratification, and short-term renal recovery. Moreover, studies that combine both FGF23 assays with KIM-1 at the time of ICU admission are scarce. To address these gaps, we measured serum iFGF23, cFGF23, and KIM-1 at ICU admission and evaluated their performance for early AKI diagnosis, AKI severity stratification, and prediction of short-term renal recovery. We aimed to delineate their respective—and potentially complementary—roles in AKI detection and short-term outcome assessment in critically ill adults.

## Methods

### Participants

Patients admitted to the ICU of Henan Provincial People’s Hospital between March 1, 2021, and June 30, 2021 were recruited. Inclusion criteria included patients aged ≥ 18 years with complete admission and discharge records and a hospital stay of more than 24 h. Exclusion criteria comprised a diagnosis of AKI prior to or at the time of ICU admission, as well as patients with chronic kidney disease (CKD) stages 3–5, defined as kidney damage or estimated glomerular filtration (eGFR) of less than 60 mL/min per 1.73 m^2^ for at least 3 months, or a documented diagnosis.

This research received approval from the Medical Ethics Committee of Henan Provincial People’s Hospital (document number 2020-206). All patients signed written informed consent at enrollment.

### Definitions and outcomes

The diagnosis and staging of AKI were determined according to the guidelines issued by the Kidney Disease Improving Global Outcomes (KDIGO) Work Group. This definition was applied irrespective of clinical judgment or other clinical parameters, focusing exclusively on the standardized laboratory criteria. Because hourly urine output data were incomplete for some patients, only Scr criteria were applied. Patients were divided into two groups, the AKI and non-AKI groups, based on the occurrence of AKI within the first week of ICU admission. Following commonly used methods in previous studies [13,14], patients with stage 1 AKI were classified as the mild AKI group, whereas those with stages 2–3 AKI were classified into the severe AKI group.

Patients were prospectively followed through the index hospitalization and classified according to renal recovery status at discharge. The recovery group comprised patients who achieved dialysis-free status at discharge with Scr levels within 10% of the baseline, consistent with prior studies of AKI recovery [15,16]. The non-recovery group included those with persistently elevated Scr levels or ongoing renal replacement therapy at discharge. Patients who died before discharge and did not meet the recovery definition were classified in the non-recovery group.

Baseline Scr was defined as the most recent value obtained within one year prior to ICU admission (using the median if multiple values were available), sourced from hospital records or outpatient laboratory results. For patients without a pre-admission measurement, the Scr value at ICU admission (prior to any signs of AKI) was used as the baseline value.

### Clinical data

At ICU admission, we recorded demographics (age and sex); comorbidities (hypertension, diabetes, cardiovascular disease, tumor, and CKD); and baseline kidney function (baseline Scr and eGFR). Laboratory variables at admission included hemoglobin, C-reactive protein, phosphate, and calcium. Peak Scr was defined as the highest Scr value recorded during the ICU hospitalization.

### Biomarker sampling and assays

Upon admission to the ICU ward, 4 mL of peripheral venous blood was drawn and subsequently centrifuged at 3000 × g for 10 min. Serum samples were stored at −80 °C for 3–6 months. Aliquoting was used to avoid repeated freeze-thaw cycles; each aliquot was thawed once and assayed immediately. Serum levels of iFGF23, cFGF23, and KIM-1 were quantified using ELISA kits in line with the manufacturers’ instructions. The ELISA kits for FGF23 (iFGF23: 60–6600; cFGF23: 60–6100) were purchased from Quidel, and those for KIM-1 (E-EL-H6029) were purchased from Elabscience.

### Sample size estimation

Sample size estimation was conducted using PASS (version 2025). Based on prior epidemiological data indicating that the incidence of AKI in ICU patients exceeds 50%, and literature reporting an expected sensitivity and specificity of approximately 75% for serum FGF23 and KIM-1 in diagnosing AKI, we performed a two-sided calculation with α = 0.05 and power (1–β) = 0.90. PASS estimated a minimum of 42 participants per group (AKI and non-AKI), for a total of 84 subjects required.

### Statistical analysis

SPSS version 31.0 was used for statistical analysis. Categorical variables were expressed as frequencies and percentages, while continuous variables were presented as means ± standard deviations or medians with interquartile ranges. Group differences in continuous variables were analyzed using the independent samples t-test or Wilcoxon rank-sum test; for categorical variables, the chi-square test or Fisher’s exact test was used. Receiver operating characteristic (ROC) curve analysis was employed to evaluate the diagnostic precision of circulating FGF23 and KIM-1 in identifying AKI, along with their ability to predict the severity of AKI and the recovery of renal function. Areas under the ROC curve (AUCs) with 95% confidence intervals (CIs) were reported; optimal cutoffs were determined by maximizing Youden’s J. We compared AUCs pairwise using DeLong’s test. All tests were two-sided, with *p* < 0.05 considered statistically significant. All biomarker measurements and clinical variables for the primary analyses were complete; therefore, no imputation was performed.

## Results

### Patient characteristic

This study involved 96 patients in total. Among these, 51 were classified into the AKI group, with an average age of 58.98 ± 17.89 years, comprising 33 males and 18 females. In the non-AKI group, there were 45 patients, with an average age of 58.38 ± 18.32 years, including 21 males and 24 females. The AKI and non-AKI groups did not differ significantly in terms of age, sex, medical history, levels of C-reactive protein, hemoglobin, or serum calcium (*p* > 0.05). The main causes of AKI were sepsis (45.10%), ischemic/hypoperfusion (25.49%), acute interstitial nephritis (AIN, 5.88%), multiple organ dysfunction syndrome (MODS, 9.80%), and other etiologies (13.73%). The AKI group showed significantly elevated baseline Scr, peak Scr, and phosphorus levels and a significantly lower baseline eGFR compared with the non-AKI group (*p* < 0.001) (Table 1). Of note, the CKD category in Table 1 reflects CKD stages 1–2 only; patients with CKD stage ≥ 3 were excluded.

**Table 1.** Demographic and clinical characteristics of AKI and non-AKI group.

Variable	Non-AKI (*n* = 45)	AKI (*n* = 51)	*P*
Male/Female	21/24	33/18	0.075
Age (years)	58.38 ± 18.32	58.98 ± 17.89	0.871
Medical history, n (%)			
Hypertension	14 (31.10%)	20 (39.20%)	0.407
Diabetes	4 (8.90%)	12 (23.50%)	0.055
Cardiovascular disease	17 (37.80%)	12 (23.50%)	0.129
Tumor	4 (8.90%)	7 (13.70%)	0.458
CKD	3 (6.70%)	5 (9.80%)	0.853
Baseline eGFR [mL/(min × 1.73 m^2^)]	101.61 (87.47–119.31)	86.09 (64.18–105.44)	0.005
Baseline Scr (μmol/L)	50.00 (39.00–69.00)	80.00 (62.00–95.00)	<0.001
Peak Scr (μmol/L)	50.00 (45.00–70.50)	128.00 (87.00–256.00)	<0.001
C-reactive protein (mg/L)	45.63 (14.25–120.41)	39.97 (18.11–147.71)	0.597
Hemoglobin (g/L)	108.36 ± 28.41	102.25 ± 28.63	0.298
Calcium (mmol/L)	2.09 ± 0.19	2.12 ± 0.21	0.483
Phosphorus (mmol/L)	1.11 ± 0.26	1.54 ± 0.72	<0.001
iFGF23 (pg/mL)	5.48 (5.07–8.11)	13.99 (5.28–51.29)	0.006
cFGF23 (RU/mL)	24.70 (14.23–51.07)	89.25 (48.45–441.13)	<0.001
KIM-1 (pg/mL)	36.72 (21.53–72.23)	224.90 (119.55–562.09)	*<*0.001
Cause of AKI (n, %)			
sepsis		23 (45.10%)	
ischemic/hypoperfusion		13 (25.49%)	
AIN		3 (5.88%)	
MODS		5 (9.80%)	
other		7 (13.73%)	
Follow-up duration (days)		8.00 (5.00–17.00)	

AKI, acute kidney injury; CKD, chronic kidney disease; eGFR, estimated glomerular filtration; Scr, serum creatine; iFGF23, intact fibroblast growth factor 23; cFGF23, C-terminal fibroblast growth factor 23; KIM-1, kidney injury molecule 1; AIN, acute interstitial nephritis; MODS, multiple organ dysfunction syndrome.

The AKI group also exhibited significantly increased serum concentrations of iFGF23 [13.99 (5.28–51.29) vs 5.48 (5.07–8.11), *p* = 0.006] and cFGF23 [89.25 (48.45–441.13) vs 24.70 (14.23–51.07), *p* < 0.001] compared to those without AKI. Moreover, the AKI group had significantly higher serum KIM-1 levels than the non-AKI group [224.90 (119.55–562.09) vs 36.72 (21.53–72.23), *p* < 0.001] (Table 1).

### FGF23 and KIM-1 for AKI prediction

**Figure 1.**
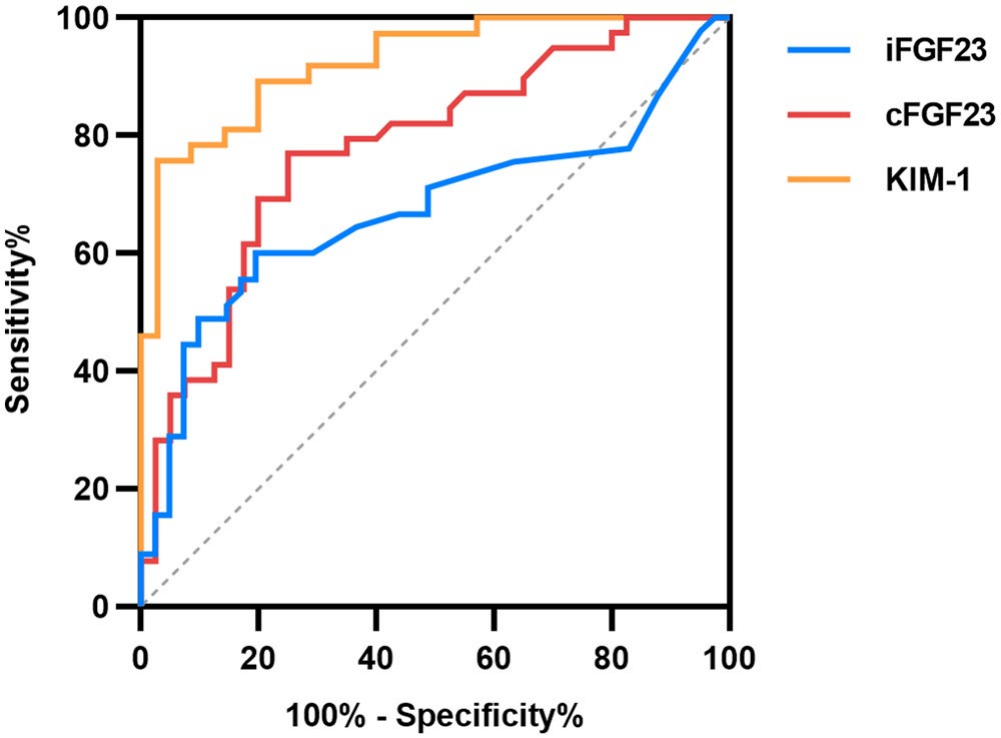
ROC curves for the prediction of AKI in critically ill patients.

The ability of the biomarkers to diagnose AKI was evaluated using ROC curve analysis, as shown in Table 2 and Figure 1. Serum cFGF23 showed significant predictive value for AKI (*p* < 0.001), with an AUC = 0.779 (95% CI 0.676–0.881), sensitivity 76.92%, specificity 75.00%, and a cutoff value of 45.23 RU/mL. Serum iFGF23 also indicated predictive potential for AKI, AUC = 0.672 (95% CI 0.554–0.789) (*p* < 0.001), with 60.00% sensitivity, 80.49% specificity, and a cutoff point of 8.93 pg/mL. Serum KIM-1 exhibited excellent predictive value for AKI (*p* < 0.001), with an AUC = 0.924 (95% CI 0.866–0.983), sensitivity 75.68%, specificity 97.14%, and a cutoff value of 125.20 pg/mL. DeLong comparisons confirmed that KIM-1 outperformed cFGF23 (*p* = 0.004) and iFGF23 (*p* < 0.001), whereas the difference between cFGF23 and iFGF23 was not significant (*p* = 0.134).

**Table 2.** ROC analyses values for serum biomarkers to predict AKI.

Variable	AUC	Cutoff value	Sensitivity	Specificity	95%CI	*P*
iFGF23	0.672	>8.93	60.00	80.49	0.554-0.789	0.006
cFGF23	0.779	>45.23	76.92	75.00	0.676-0.881	<0.001
KIM-1	0.924	>125.20	75.68	97.14	0.866-0.983	<0.001

ROC, receiver operating characteristic; AUC, area under the curve; iFGF23, intact fibroblast growth factor 23; cFGF23, C-terminal fibroblast growth factor 23; KIM-1, kidney injury molecule 1.

### FGF23 and KIM-1 for severe AKI prediction

Among the 51 patients suffering from AKI included in this study, 26 were diagnosed with stage 1 AKI, 7 with stage 2, and 18 with stage 3. Patients with stage 1 AKI were classified into the mild AKI group (*n* = 26), while those with stages 2–3 AKI were grouped into the severe AKI group (*n* = 25). No notable differences in gender, age, baseline Scr, baseline eGFR, or serum KIM-1 levels were found between the mild and severe AKI groups (*p* > 0.05). However, individuals with severe AKI demonstrated significantly elevated serum concentrations of iFGF23 [51.29 (11.86–404.63) vs 6.70 (4.67–20.07), *p* = 0.001] and cFGF23 [195.81 (84.42–532.13) vs 67.38 (20.14–249.09), *p* = 0.009] compared to those with mild AKI (Table 3).

**Table 3.** Demographic and clinical characteristics of patients in mild AKI group and severe AKI group.

Variable	Mild AKI (*n* = 26)	Severe AKI (*n* = 25)	*P*
Male/Female	13/13	17/8	0.192
Age (years)	62.27 ± 14.43	55.56 ± 20.63	0.065
Medical history, n (%)			
Hypertension	8 (30.80%)	12 (48.00%)	0.208
Diabetes	6 (23.10%)	6 (24.00%)	0.938
Cardiovascular disease	5 (19.20%)	7 (28.00%)	0.460
Tumor	3 (11.50%)	4 (16.00%)	0.955
CKD	2 (7.70%)	3 (12.0%)	0.963
Baseline eGFR [mL/(min × 1.73 m^2^)]	86.09 (64.97–109.15)	83.03 (66.67–99.11)	0.447
Baseline Scr (μmol/L)	75.00 (60.00–89.00)	85.50 (68.50–102.50)	0.117
Peak Scr (μmol/L)	96.50 (77.00–173.00)	219.00 (110.50–419.50)	0.003
C-reactive protein (mg/L)	42.78 (19.58–174.50)	35.70 (17.91–111.28)	0.434
Hemoglobin (g/L)	101.08 ± 24.19	103.48 ± 33.09	0.768
Calcium (mmol/L)	2.10 ± 0.19	2.13 ± 0.24	0.617
Phosphorus (mmol/L)	1.22 ± 0.41	1.87 ± 0.82	0.001
iFGF23 (pg/mL)	6.70 (4.67–20.07)	51.29 (11.86–404.63)	0.001
cFGF23 (RU/mL)	67.38 (20.14–249.09)	195.81 (84.42–532.13)	0.009
KIM-1 (pg/mL)	173.16 (78.21–558.92)	386.43 (172.93–596.60)	*0.075*

AKI, acute kidney injury; CKD, chronic kidney disease; eGFR, estimated glomerular filtration; Scr, serum creatine; iFGF23, intact fibroblast growth factor 23; cFGF23, C-terminal fibroblast growth factor 23; KIM-1, kidney injury molecule 1.

**Figure 2.**
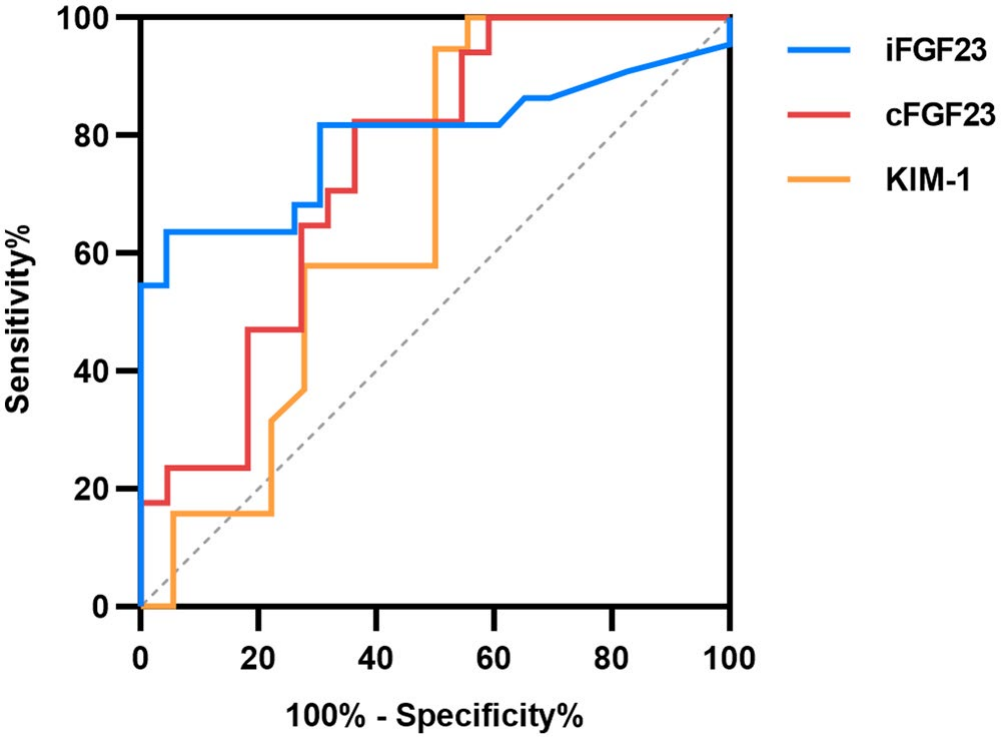
ROC curves for the prediction of severe AKI in critically ill patients.

ROC curve analysis showed that serum iFGF23 had significant discriminatory power for severe AKI (*p* < 0.001), with an AUC of 0.793 (95% CI 0.650–0.935), sensitivity 63.64%, specificity 95.65%, and a cutoff value of 27.78 pg/mL. Similarly, serum cFGF23 also predicted severe AKI (*p* = 0.009), with an AUC of 0.746 (95% CI 0.593–0.899), sensitivity 82.35%, specificity 59.09%, and a cutoff value of 82.94 RU/mL. In contrast, serum KIM-1 was not a significant predictor of severe AKI (*p* = 0.076) (Table 4, Figure 2). DeLong’s test indicated no significant difference between iFGF23 and cFGF23 in predicting severe AKI (*p* = 0.958).

**Table 4.** ROC analyses values for serum biomarkers to predict severe AKI.

Variable	AUC	Cutoff value	Sensitivity	Specificity	95%CI	*P*
iFGF23	0.793	>27.78	63.64	95.65	0.650-0.935	<0.001
cFGF23	0.746	>82.94	82.35	59.09	0.593-0.899	0.009
KIM-1	0.671	>140.00	97.74	50.00	0.487-0.855	0.076

ROC, receiver operating characteristic; AUC, area under the curve; iFGF23, intact fibroblast growth factor 23; cFGF23, C-terminal fibroblast growth factor 23; KIM-1, kidney injury molecule 1.

### FGF23 and KIM-1 for outcome prediction

**Figure 3.**
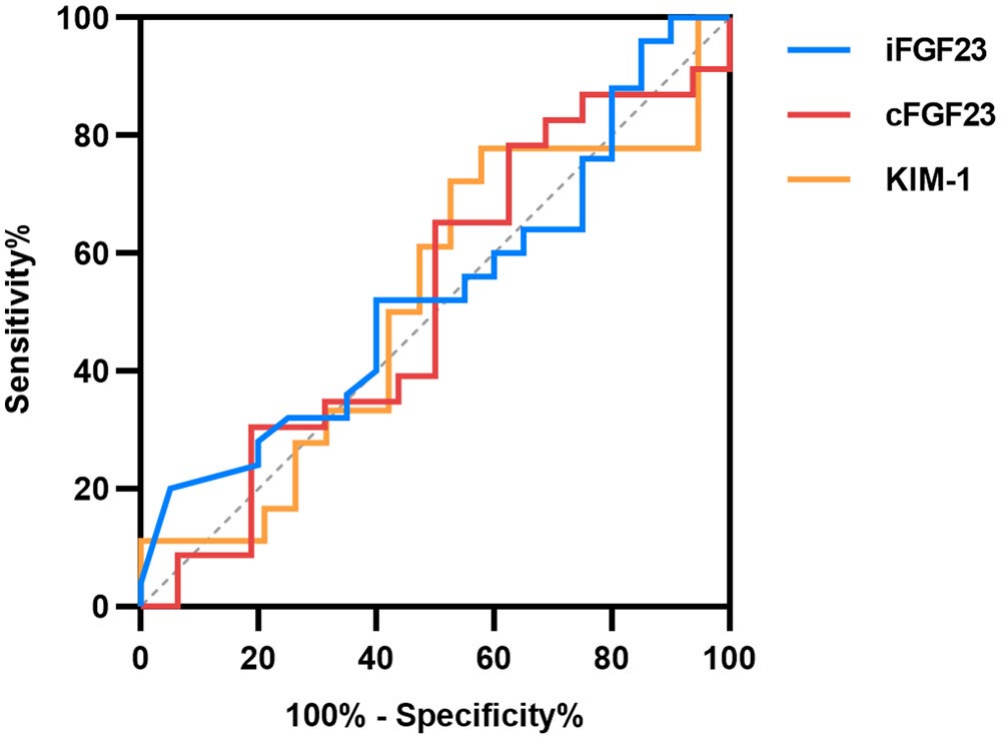
ROC curves for the prediction of renal function recovery in AKI patients.

The median follow-up duration was 8.00 (5.00–17.00) days for AKI patients overall. At the end of the observation period, 23 patients (45.10%) experienced recovery of renal function, while 28 patients (54.90%) did not. Baseline serum levels of iFGF23, cFGF23, and KIM-1 did not differ significantly between the groups with and without renal function recovery (all *p* > 0.05) (Table 5). ROC analysis for renal function recovery was performed in the 51 patients who developed AKI and had complete follow-up. None of the biomarkers—iFGF23, cFGF23, or KIM-1—showed significant predictive ability (all *p* > 0.05, Figure 3).

**Table 5.** Comparison of serum biomarkers in patients with and without renal function recovery.

Variable	Recovered (*n* = 23)	Non-recovered (*n* = 28)	*P*
iFGF23	15.21 (5.58–50.89)	11.56 (4.87–56.77)	0.689
cFGF23	105.09 (24.50–346.92)	89.25 (53.82–449.60)	0.842
KIM-1	327.61 (102.67–596.60)	220.18 (122.64–585.96)	0.855

ROC, receiver operating characteristic; AUC, area under the curve; iFGF23, intact fibroblast growth factor 23; cFGF23, C-terminal fibroblast growth factor 23; KIM-1, kidney injury molecule 1.

## Discussion

This study evaluated the predictive value of serum FGF23 (intact and C-terminal) and KIM-1 for AKI in critically ill adults. All three biomarkers were elevated in patients with AKI and predicted AKI occurrence, with KIM-1 demonstrating the strongest diagnostic discrimination. By contrast, iFGF23 and cFGF23—but not KIM-1—were associated with severe AKI, suggesting complementary roles: KIM-1 for early detection and FGF23 for severity stratification. None of the biomarkers predicted short-term renal recovery.

FGF23, a key regulator of mineral metabolism, increases in CKD and has been associated with cardiovascular disease [17,18]. Recent studies have reported marked increases of circulating FGF23 in AKI with potential diagnostic utility, although results have been mixed across settings and assays [19,20]. In a cohort study of 79 ICU patients, researchers discovered that cFGF23, but not iFGF23, was notably elevated in AKI, and cFGF23 levels > 136 RU/mL could predict AKI with an AUC of 0.81 (sensitivity 83%, specificity 82%) [21]. By contrast, Shaker et al. [22] found that iFGF23 could forecast the development of AKI in patients undergoing cardiac surgery, whereas a pediatric ICU cohort showed no relationship between serum FGF23 and AKI [23]. Our research focused on adult ICU patients and demonstrated that both serum iFGF23 and cFGF23 were predictive of AKI development. Although cFGF23 showed a numerically higher AUC for diagnosis, the two assays (cFGF23 and iFGF23) were statistically similar. These data suggest that FGF23 may offer early diagnostic value for AKI.

KIM-1 reflects proximal tubular epithelial injury and has been reported as a biomarker of AKI [24]. In a study of 45 patients undergoing cardiac surgery, Khreba et al. found that urinary KIM-1 > 1.9 ng/mg could specifically diagnose AKI with AUC of 0.715 (sensitivity 48%, specificity 94%) [25]. In our ICU cohort, serum KIM-1 predicted AKI and showed greater diagnostic discrimination than both iFGF23 and cFGF23, consistent with a prospective sepsis cohort in which KIM-1 showed a higher AUC than FGF23 [26]. Collectively, these data suggest that serum KIM-1 may exhibit superior diagnostic performance to FGF23 in diagnosing AKI at an early stage.

With respect to severity, patients with severe AKI had higher serum iFGF23 and cFGF23, and both assays could predict severe AKI. This finding is consistent with prior cardiac surgery cohorts in which cFGF23 was associated with the risk of severe AKI [19]. Although urinary NGAL and KIM-1 have also been associated with severity, their AUCs were lower than those of cFGF23 in their study [19]. In our cohort, serum KIM-1 did not predict severe AKI. Conversely, several studies have reported that KIM-1 predicts severe AKI. Arthur et al. [27] reported excellent discrimination for AKIN stage 3 or death using the combination of IL-18 and KIM-1 (AUC = 0.93) among postoperative patients initially at AKIN stage 1. The discrepancies may be explained by different populations (medical vs surgical ICU), baseline comorbidities, sampling time points, and underlying pathophysiology.

None of the three biomarkers predicted short-term renal recovery in our cohort. This may be due to the limited sample size (51 AKI patients) and the short duration of inpatient follow-up. Moreover, recovery from AKI is multifactorial and influenced by systemic factors (comorbidities, hemodynamics, inflammation, and therapies), so a single early time-point biomarker measurement may be insufficient. Several clinical studies have linked higher FGF23 concentrations to adverse outcomes in AKI. Sakan et al. [28] enrolled 121 adult patients with AKI following major surgery and reported that FGF23 > 630 RU/mL was related to poor renal function recovery. Wu et al. [29] enrolled 257 AKI patients and found that plasma cFGF-23 exhibited superior predictive capability for patient outcomes compared to NGAL, KIM-1, and Scr. Among CKD patients with superimposed AKI requiring renal replacement therapy, cFGF23 discriminated 90-day mortality better than NGAL [30]. By contrast, in a cohort of pediatric patients with acute respiratory distress syndrome, Hanudel et al. [31] found that only cFGF23, but not iFGF23, was associated with 60-day mortality. Experimental data suggest that FGF23 may exert protective effects in ischemia-reperfusion injury; preconditioning with FGF23 enhanced tubular regeneration and vascular repair, thereby attenuating tissue injury [32]. Evidence for KIM-1 is similarly heterogeneous. While some studies reported that higher urinary or plasma KIM-1 associated with worse outcomes [33,34], other investigations have found no independent prognostic value for KIM-1 or only modest discrimination after adjustment for clinical covariates considered [35,36]. Taken together, the prognostic meaning of FGF23 (intact vs C-terminal) and KIM-1 appears context-dependent and may vary by population, timing, specimen matrix, assay, and covariate adjustment. Standardized, multicenter studies with serial measurements, harmonized assays, and extended post-discharge follow-up are warranted to determine whether these markers are causal drivers of poor outcomes, compensatory responses, or both.

Our findings support a complementary biomarker strategy in the ICU setting. KIM-1 appears best suited to early recognition of AKI, whereas FGF23 (both iFGF23 and cFGF23) may enrich for severity risk once injury is established. Implementation should be pragmatic and integrated with clinical variables and existing risk scores.

This study has several limitations. First, the single-center design with limited sample size may restrict generalizability. Second, biomarkers were measured only at ICU admission, the absence of serial measurements precluded assessment of temporal trajectories during AKI evolution and recovery. Third, the short follow-up period focused solely on in-hospital outcomes, so we could not capture post-discharge trajectories. Finally, we did not conduct multivariable logistic regression because the number of outcome events was insufficient for stable adjustment without overfitting; thus, residual confounding cannot be excluded. Nonetheless, prior studies have reported associations of FGF23 and KIM-1 with AKI incidence/severity across diverse settings after adjustment for clinical factors [9,20,37], supporting the biological plausibility of our findings.

In conclusion, elevated serum levels of iFGF23, cFGF23, and KIM-1 demonstrated significant diagnostic value for AKI onset in ICU patients, with KIM-1 exhibiting the highest specificity. Both iFGF23 and cFGF23 demonstrated significant discrimination for severe AKI, whereas KIM-1 lacked utility in severity stratification. None of the biomarkers predicted in-hospital renal recovery. These findings support a complementary biomarker strategy in which KIM-1 aids early recognition, whereas FGF23 assays inform severity risk. Multicenter studies incorporating serial sampling, harmonized assays, longer follow-up, and multivariable adjustment are needed to define how KIM-1 and FGF23 can be combined with clinical data to improve AKI detection, risk stratification, and outcome prediction.

## Data Availability

All data are also available upon request.
